# Walk Score and Neighborhood Walkability: A Case Study of Daegu, South Korea

**DOI:** 10.3390/ijerph20054246

**Published:** 2023-02-27

**Authors:** Eun Jung Kim, Suin Jin

**Affiliations:** Department of Urban Planning, Keimyung University, 1095 Dalgubeol-daero, Dalseo-gu, Daegu 42601, Republic of Korea

**Keywords:** Walk Score, neighborhood walkability, built environment, Daegu, South Korea

## Abstract

Walking is a popular physical activity that helps prevent obesity and cardiovascular diseases. The Walk Score, which measures neighborhood walkability, considers access to nine amenities using a geographic information system but does not deal with pedestrian perception. This study aims to (1) examine the correlation between access to each amenity, an individual component of the Walk Score, and perceived neighborhood walkability and (2) investigate the correlation with the perceived neighborhood walkability by adding variables of pedestrian perception to the existing Walk Score components. This study conducted a survey with 371 respondents in Daegu, South Korea, between 12 October and 8 November 2022. A multiple regression model was used to examine the correlations. The results showed no association between perceived neighborhood walkability and the individual component of the Walk Score. As variables of environmental perception, the fewer hills or stairs, the more alternative walking routes, the better separation between road and pedestrians, and the richer the green space, the more people perceived their neighborhood as walkable. This study found that the perception of the built environment had a more substantial influence on perceived neighborhood walkability than the accessibility to amenities. It proved that the Walk Score should include pedestrian perception and quantitative measurement.

## 1. Introduction

Walking is an inexpensive and popular physical activity that helps prevent obesity and cardiovascular disease [1,2,3,4]. The advantages of walking have increased interest in a walkable environment [5,6]. The built environment plays a crucial role in determining the quality of walking, and a well-designed built environment positively affects walkability [7,8,9]. Therefore, it is essential to understand how the characteristics of the built environment are related to walking.

Various indices have been developed to measure the built environment regarding walkability [1,10,11,12]. The Walk Score—a user-friendly and convenient index—is widely used in many studies [11,13]. The index enables comparing environmental walkability among countries and is used globally [14]. Walk Score comparison between cities in Western countries and Seoul in South Korea enables intriguing research; such comparison can draw policy implications from environmental walkability differences between countries [15].

Kim et al. measured the Walk Score in Seoul and confirmed some significant correlation with pedestrian satisfaction [16]. However, this study points out that although the Walk Score is efficient as a walkability index, it is limited in its application to the Asian context. The built environment in Korea has dense and mixed land use, and amenities are oversupplied in most cities with these characteristics [17,18,19,20]. Consequently, people have easy access to amenities by walking. In this context, Korean cities can receive a high Walk Score, but explaining Korea’s walkability only with accessibility to the destinations, which constitutes the Walk Score, can cause distorted results [21]. Therefore, it is necessary to consider the additional variables required to develop the Walk Score as a walkability index suitable for the Korean context.

The Walk Score is a quantitative indicator calculated by nine types of access to amenities and two types of pedestrian friendliness [22]. However, it has shortcomings in that it does not include the pedestrians’ perception of the built environment [7]. Bereitschaft said evaluating walkability only based on the Walk Score has low accuracy since it does not consider people’s perception of the neighborhood environment, even though it has an advantage in understanding the overall neighborhood walkability [11]. Tuckel and Milczarski found that the Walk Score was associated with walking for transport but not with walking for leisure purposes. They did not examine the association between the Walk Score and the perceived neighborhood walkability, and therefore, it will be necessary to examine the relationship between them [6]. Perception of the built environment in which people live determines their walking attitude and willingness to walk, and from this perspective, it is essential to understand citizens’ perception of the built environment that affects walking [1,23,24,25].

Therefore, this study aims to (1) find out how much the nine amenity elements constituting the Walk Score are related to the perceived neighborhood walkability of the residents and (2) add variables measuring people’s awareness of the built environmental walkability to the Walk Score and examine the correlation between the perceived neighborhood walkability.

## 2. Literature Review

### 2.1. Concept of the Walk Score

The Walk Score is a free public web-based tool for measuring local walkability and is serviced in the USA, Canada, Australia, and New Zealand. The Walk Score is determined by its accessibility to nine amenities (grocery, restaurants, shopping, coffee shops, banks, parks, schools, books, and entertainment) for each address [22]. When the facility is within 0.25 miles, it obtains a full score using a distance decay function. The score decreases as the distance increases, providing a score for the amenity within up to 1.5 miles [26].

Based on this, scores are generated according to the network distance from the address to the destination and the facility’s weight. Each amenity obtains a different weight, and the considered number of facilities differs (Table 1). The weights and the number of facilities were determined based on previous studies of walkability. Then, it multiplies 6.67 by each amenity’s weight and aggregates the numbers to obtain a normalized score from 0 to 100. Meanwhile, penalties based on average block length and intersection density, considered pedestrian friendliness, deduct scores by 1–10% [22]. The Walk Score is calculated in this method, and the scores are divided into five tiers: 0–24 points are “car-dependent”, 25–49 points are “somewhat car-dependent”, 50–69 points are “somewhat walkable”, 70–89 points are “very walkable”, and 90–100 points are “walker’s paradise” [26].

### 2.2. Built Environmental Factors and Walking

Several studies have found a significant association between the built environment and walking [24,27,28,29,30,31,32,33,34,35,36,37,38,39]. This study classified built environmental factors that affect walking into four categories: convenience, connectivity, safety, and comfort. First, on the convenience of the built environment, Kim et al. and Lee et al. investigated the factors affecting pedestrian volume and satisfaction in Seoul and found that wider sidewalks increase pedestrian volume and satisfaction, while steep roads negatively affect them [24,33]. Herrmann-Lunecke et al. examined how pedestrians’ perception of the built environment affects the walking experience in Santiago, Chile [30]. This study showed that pedestrians are happier when the sidewalk is wider, while narrow sidewalks invoke anger and fear. Similarly, Zumelzu Scheel et al. investigated pedestrians’ perceptions of the built environment in southern Chile and confirmed that wider sidewalks in good condition promote walking [39]. These studies show that convenience is related to the ease and efficiency of walking and making people willing to walk. Therefore, factors that measure convenience may include the presence of various facilities, sidewalk width, sidewalk condition, hills and stairs, and pedestrian shelters. 

Second, on connectivity, Adkins et al. analyzed how urban design characteristics affect the perception of walking environment attractiveness [27]. This study showed that walking environment attractiveness increases with better pedestrian connectivity. Liao et al. examined factors influencing walking time for people in Taiwan [34]. The study showed that if the roads are well connected, people are likelier to walk more than 150 min weekly. Furthermore, Ferrari et al. examined the relationship between the perceived built environment of neighborhoods and walking and cycling in eight Latin American countries [29]. As a result, the study found that people were more likely to choose walking for transport when there were more alternative routes in the neighborhood. Meanwhile, Nag et al. identified that well-connected pedestrian road without obstacles promotes pedestrian satisfaction [35]. Connectivity is a factor evaluating whether walking is uninterrupted and whether the road network is well connected. Specifically, more alternative routes, better sidewalk connections, and fewer pedestrian obstacles can be the factors of connectivity and will further increase pedestrian walking. 

Third, Yu et al., Ariffin and Zahari, and Oyeyemi et al. studied the safety factor of the built environment [28,36,37]. Yu et al. investigated how the elements of perceived neighborhood walkability are related to well-being and loneliness among older adults in Hong Kong [37]. The study found that traffic safety is significantly associated with well-being, which decreases when pedestrians face difficulties with walking due to heavy traffic. Meanwhile, Ariffin and Zahari examined the built environmental factors that can promote walking behavior in Malaysia [28]. This study showed that reducing the risk of crime motivates people to walk, emphasizing the importance of safety awareness to encourage walking. Oyeyemi et al. investigated the relationship between older adults’ sedentary time and attributes of the neighborhood environment in Nigeria [36]. The results showed that lacking safety from crime is likely to increase older adults’ sedentary time. The safety category measures whether pedestrians can walk safely from traffic collisions and crime. Accordingly, variables such as street segregation, crosswalk and traffic lights, and traffic volume can be used as the safety factors from traffic collisions, and security facilities (CCTV, streetlights, etc.) may be employed as safety factors from crime.

Fourth, Zhang et al. investigated the relationship between older adults’ frequency and duration of the walking trip and the built environment in the Zhongshan Metropolitan area, China [38]. The study found that older adults were more encouraged to walk when the percentage of green space land use was higher. Lee et al. examined the correlation between the neighborhood environment variable and neighborhood satisfaction [31]. The result showed that perceived aesthetics, such as trees in the neighborhood, no trash, attractive architecture, and natural scenery, positively correlated with neighborhood satisfaction. In another study, Lee et al. examined the factors affecting pedestrian satisfaction according to land use and road type [32]. Results showed that green space positively influenced pedestrian satisfaction, and clean streets in the commercial district also increased satisfaction. The comfort factor is how pleasant the built environment is to walk, and it measures pedestrians’ perception of green space, noise levels, etc. More green spaces and natural scenery, cleaner streets, less odor and smoke, and lower noise levels can be comfort factors that promote pedestrian walking.

As we reviewed above, the built environmental factors that affect walking were summarized into four categories: convenience, connectivity, safety, and comfort. According to the previous studies examined, the variables of walkable built environments that can be investigated in the survey questionnaire of this study were selected as follows: (1) convenience: various facilities, sidewalk width, sidewalk conditions, hills and stairs, and pedestrian shelters, (2) connectivity: multiple alternative routes, sidewalk connection, and pedestrian obstacles, (3) safety: pedestrian segregation, crosswalk and traffic lights, traffic volume, and security facilities, (4) comfort: green spaces, natural scenery, street cleanness, odor and smoke, and noise level. We will use 17 items from four categories as the independent variables of the built environmental perceptions in this study. 

## 3. Materials and Methods

### 3.1. Research Area and Data Collection

**Figure 1:**
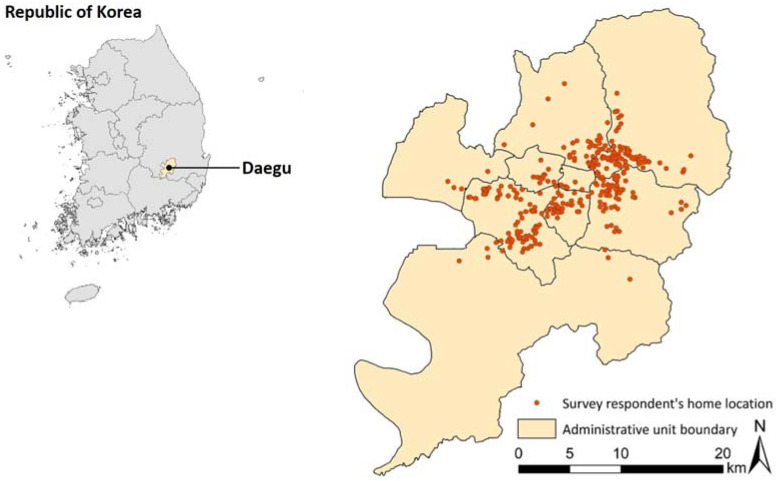
Research area and the survey respondent’s location.

This study covered the city of Daegu, located in the southeastern part of South Korea (Figure 1). Daegu is 883.7 km^2^ wide and had a population of 2,385,412 as of 2021 [40]. This study used survey data from the larger project (Healthy Walking Project) and was conducted from 12 October to 8 November 2022. This study was approved by the institutional review board of the research team and surveyed individuals aged 18 or older to examine the awareness of neighborhood walkability in the built environment. The questionnaire was distributed to a total of 487 people, and 371 valid responses were used for analysis in this study.

### 3.2. Measure

#### 3.2.1. Dependent Variable: Perceived Neighborhood Walkability

The dependent variable of this study, the perceived walkability of the neighborhood, was collected through a survey. The respondents evaluated the degree of the walkability of their neighborhood between 0 and 100 points to the question “How good is the walkability of your neighborhood?”. The average of the perceived neighborhood walkability was 76.10 (SD = 17.57).

#### 3.2.2. Independent Variables

- Access to nine types of amenities used in the Walk Score

**Figure 2:**
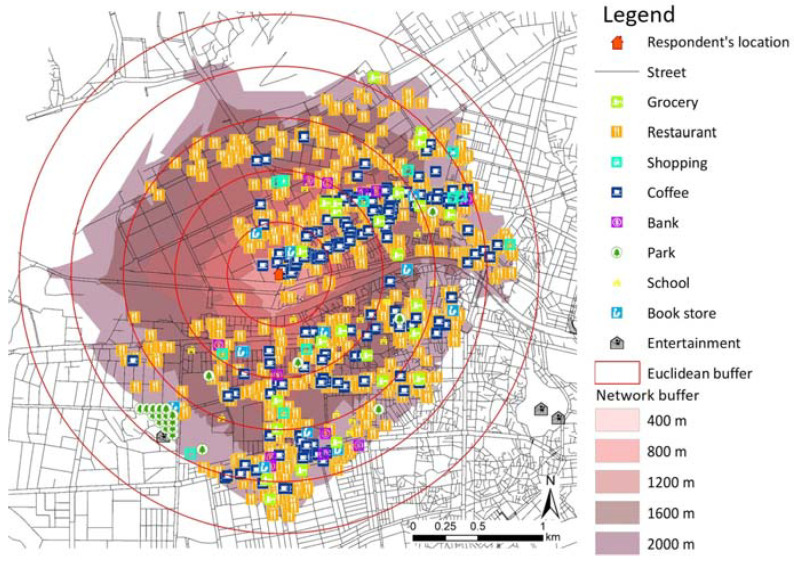
Nine types of amenities within walking distance from the respondent’s location.

This study used the weighted accessibility values to each amenity comprising the Walk Score as independent variables. As shown in Table 1, grocery and restaurants have a weight of 3, shopping and coffee shop have a weight of 2, and the remaining five amenities have a weight of 1. For this reason, the amenity accessibility values with the weight are slightly different for each amenity in Table 2. Meanwhile, data on five amenities, grocery, restaurants, shopping, coffee shop, and entertainment, were collected from D-Data Hub [41]. Data on banks were obtained from the Financial Supervisory Service of Korea by requesting location data. Data on parks and schools were collected from Road Name Address [42], address-based industry support services, and data on Books from BigData MarketC [43]. Moreover, the average block length and intersection density related to pedestrian friendliness are factors deducting scores. According to the Walk Score criteria, the average block length is less than 120 m, and the intersection density is higher than 200 at the locations of all respondents in this study. Therefore, when calculating the average block length and intersection density within the 400 m network buffer based on the respondents’ address in this study, there was no deduction at all points, and this study did not need to consider the two pedestrian friendliness factors. Accessibility with the weights of the individual amenities constituting the Walk Score was calculated using ArcGIS 10.5 (Esri, Redlands, CA, USA). Figure 2 shows the locations of nine amenities, which are the Walk Score components, within walking distance from the respondent’s home.

- Perception of the built environment

The perception variable that evaluates neighborhood walkability in the built environment was selected based on the literature review, and data were collected through the survey. It consists of 17 items under four categories (Table 2). All the perception variables were measured with a 5-point Likert scale, from strongly disagree (1), disagree (2), neutral (3), agree (4), and strongly agree (5). 

#### 3.2.3. Control Variables

As control variables, several individual characteristics of the respondents, such as gender, age, and weekly minutes of walking, were used, and the data were collected through the survey. For weekly minutes of walking, the number of walking days per week is multiplied by the average walking time (as minutes). In the descriptive statistics, 37.5% of the respondents were men, and 62.5% were women. The average age of respondents was 34.80 (SD = 14.49), and the weekly minutes of walking was 161.62 (SD = 115.74). 

### 3.3. Statistical Analysis

This study conducted a regression analysis to investigate the relationship among perceived neighborhood walkability, the Walk Score, and built environment awareness. This study used SPSS 27 (IBM Corporation, Armonk, NY, USA) software for the analysis.

## 4. Results

Table 3 shows the results of multiple regression analysis for the perceived neighborhood walkability. Model 1 considered only the Walk Score’s nine amenities, and Model 2 included additional variables that measure the perception of the built environment. The results are as follows. First, in Model 1, the perceived neighborhood walkability was higher when the school had better accessibility, and the other eight amenities did not significantly associate with the perceived neighborhood walkability. In this context, Model 2 found that only access to banks was a significant variable related to the perceived neighborhood walkability among amenities consisting of the Walk Score. These results show that the accessibility to amenities is not significantly correlated to perceived neighborhood walkability. In other words, the Walk Score alone cannot explain the degree to which the pedestrians feel good about walking. It suggests that additional variables are required along with accessibility to amenities.

Second, this study examined the variables measuring the perception of the built environment in Model 2. Excluding the items with multicollinearity problems, 6 out of 17 variables were analyzed. The analysis confirmed that the perceived neighborhood walkability decreases as people feel uncomfortable with steep roads and stairs, similar to previous studies [24,44,45]. In addition, multiple alternative routes correlated statistically with perceived neighborhood walkability at 0.001 level. It means pedestrians think the walkability is higher when more alternative routes are available. This result is similar to a previous study showing that alternative routes to access the destination are likely to promote walking [29]. Meanwhile, pedestrians generally thought their neighborhood was more walkable with clear segregation between pedestrians and vehicles, and previous studies also confirmed this finding [27,28,30,38,46,47,48]. In the case of green spaces, there was a statistically significant correlation with the perceived neighborhood walkability at the significance level of 0.001. Pedestrians think the walkability is higher when they feel the neighborhood has visually rich green spaces, and this is similar to previous studies that proved that green spaces positively influence walking [24,27,49,50]. Traffic volume, odor, and smoke on the road did not show a statistically significant association with the perceived neighborhood walkability.

Third, the respondents’ gender, age, and weekly minutes of walking did not have a statistically significant correlation with neighborhood walkability. Xiao et al. found that women and older adults are more likely to walk [51]. In addition, a similar study found that the average number of walks per week positively affected emotional health but did not show significant results related to physical activity [52]. Although previous studies have shown that individual characteristics are significantly associated with walking, this study shows an insignificant association between individual characteristics and perceived neighborhood walkability.

## 5. Discussion

Several studies prove that the built environment has a significant relationship with walking, and interest in creating a walkable environment has increased accordingly. It used the Walk Score walkability index to understand neighborhood walkability. However, Walk Score has limitations as a walkability index in dense countries such as South Korea. Moreover, the Walk Score misses a qualitative indicator to measure the perception of the built environment. Previous studies argued that if the perception of the built environment from a qualitative perspective is considered along with quantitative walkability indicators, it will better measure neighborhood walkability [6]. Therefore, this study attempted to examine whether the accessibility to the nine amenities used in the Walk Score relates to the perceived neighborhood walkability. Furthermore, this study aimed to determine the correlation with the perceived neighborhood walkability, including the variables measuring the perception of the built environment.

The discussed contents based on the research results are as follows. First, it was confirmed that the Walk Score alone, which mainly considers accessibility to the destination, does not estimate the perceived neighborhood walkability. Model 1, which examined the relationship between accessibility to amenities and perceived neighborhood walkability, showed 0.027 explanatory power. Model 2, including variables measuring the perception of the built environment, showed a relatively high explanatory power of 0.335. The increase in explanatory power in Model 2 is due to the inclusion of perception variables. It also showed that the accessibility to most destinations in the Walk Score could not explain the perceived neighborhood walkability. Therefore, the Walk Score, which focuses on amenities, does not reflect neighborhood walkability and requires additional qualitative variables.

Second, this study proved that the perception variables of walkability in the built environment in a highly dense city are significantly related to perceived neighborhood walkability. Among the perception of the built environment variables, hills and stairs hindered the perceived neighborhood walkability. Hills and stairs are the elements that disturb walking, causing detours and risk of falls. Therefore, hills and stairs can be used to evaluate convenience and safety. In particular, since older people are more vulnerable to slopes and stairs, they can be considered when evaluating walkability by age group. Third, the diversity of alternative routes increased the perceived neighborhood walkability. Pedestrians perceived it as more pedestrian-friendly when more options were given to reach their destination because they could walk the preferred route. Similarly, previous studies have also argued that areas with good connectivity provide more routes [53]. Therefore, the alternative route is expected to be a significant variable when evaluating street connectivity in the future. Fourth, pedestrian segregation increased the perceived neighborhood walkability. The sidewalk is the most basic pedestrian infrastructure and is crucial for pedestrians deciding on the walking route [49,54]. Pedestrian segregation should be considered for walkability since it promotes pedestrians’ psychological safety. Accordingly, perceptions of pedestrian segregation can be included in the safety aspect. Fifth, green spaces bring pleasantness and enhance perceived neighborhood walkability. Pedestrians walk longer and are more satisfied when walking a route with abundant green spaces and well-managed [27,55]. Moreover, green spaces can be an indicator of identifying a pedestrian-friendly environment as it is an essential factor in determining health. Therefore, not only the accessibility to the park constituting the Walk Score but also the perceived green space can be considered for the evaluation item of the pedestrian friendliness index. Moreover, since green spaces can be identified using the Normalized Difference Vegetation Index (NDVI) and Google Street View, it will be more widely applicable if they are quantitatively evaluated in future research. These results proved that the perception variable of the built environment significantly affected the individual’s perceived walkability. Therefore, it is necessary to introduce additional qualitative variables and quantitatively measured built environments to evaluate neighborhood walkability in the future.

This study’s limitations and future research direction are as follows. First, it is necessary to verify whether the results of this study can be applied to cities other than Daegu and such research in the future will be efficient in understanding walkability in South Korea. This study was conducted in a large city, Daegu, but future research on small and medium-sized cities will be effective. Second, though this study used the Walk Score only, future research can expect higher policy applicability by using other qualitative and quantitative indicators such as the Walkability Index, Pedestrian Index of the Environment, and the Neighborhood Destination Accessibility Index, including the Walk Score to examine correlation with perception of the built environment. Lastly, this study was conducted on all adult groups aged 18 or older, but it is necessary to consider especially for the elderly who have mobility difficulties. This is because the level of perceived neighborhood walkability will vary depending on age. It can be an important future study to examine the built environmental factors that affect the neighborhood walkability of the elderly with restrictions on walking [56]. Furthermore, as attempted in Hirsch and colleagues’ study, it will be possible to examine the Walk Score and the mobility of older adults [57].

Despite these limitations, this study reviewed whether the Walk Score could explain the individual’s perceived neighborhood walkability in Daegu with the high density of the built environment. It further examined the correlation between the perceived neighborhood walkability and the perception of the built environment in addition to the Walk Score. This study found that the perception of the built environment had a more substantial influence on perceived neighborhood walkability than the accessibility to amenities. It proved that the Walk Score should include pedestrian perception and quantitative measurement. It is also meaningful in that it provided evidence that the perception that people experience should be accompanied by the quantitatively measured Walk Score.

## 6. Conclusions

Promoting walking is suggested as one of the physical activities for health in public health, transportation engineering, and urban planning. This study determines whether the accessibility to the destination, which consists of the Walk Score, is related to the perceived neighborhood walkability. We added the variables of perception of the built environment to investigate the relationship between the perceived neighborhood. As a result, the Walk Score alone could not explain the perceived neighborhood walkability. Moreover, it confirmed a correlation between the perception of the built environment and perceived neighborhood walkability. Therefore, this study argues that more qualitative factors should be included and suggests additional perceptions of the built environment must be considered in Walk Score. This study is expected to contribute to developing the Walk Score to the next level. 

## Figures and Tables

**Table 1:** Walk Score algorithm.

Category	Algorithm		
	Total Count	Weight	
		Weight of *i*th Nearest Amenity ^1^	Total Weight
Grocery	1	3	3
Restaurants	10	0.75, 0.45, 0.25, 0.25, 0.225, 0.225, 0.225, 0.225, 0.2, 0.2	3
Shopping	5	0.5, 0.45, 0.4, 0.35, 0.3	2
Coffee Shops	2	1.25, 0.75	2
Banks	1	1	1
Parks	1	1	1
Schools	1	1	1
Books	1	1	1
Entertainment	1	1	1

Note: ^1^ Multiple numbers mean that multiple counts for that amenity are included. The closest and the second closest amenities of the types receive the weights of the first and second numbers, respectively, etc. Source: Walk Score Methodology [22].

**Table 2:** Measurement and descriptive statistics of survey data.

Category	Variable	Measurement	Descriptive Statistics
			Mean (SD)
*Dependent Variable*			
Perceived neighborhood walkability		Degree of walkability of the neighborhood perceived by each respondent Continuous: 0–100	76.10 (17.57)
*Independent Variables*			
Access to amenities used in the Walk Score			
Grocery		Continuous: ranging from 0 to 3	2.85 (0.41)
Restaurants			2.90 (0.26)
Shopping		Continuous: ranging from 0 to 2	0.47 (0.38)
Coffee shops			1.89 (0.27)
Banks		Continuous: ranging from 0 to 1	0.78 (0.27)
Parks			0.87 (0.20)
Schools			0.84 (0.20)
Books			0.81 (0.24)
Entertainment			0.24 (0.32)
Perception of the built environment ^1^			
Convenience	Various facilities	Various facilities (convenience store, café, etc.) are accessible on foot	4.03 (0.92)
	Sidewalk width	Sufficient width of sidewalk for comfort walking	3.49 (1.12)
	Sidewalk conditions	Sidewalk is well-paved and maintained	3.61 (0.98)
	Hills and stairs	Difficulties with walking due to hills and stairs	2.44 (1.09)
	Pedestrian shelters	Rest facilities such as benches are found	3.29 (1.17)
Connectivity	Multiple alternative routes	Various routes are available to reach a certain destination	3.77 (0.98)
	Sidewalk connection	Sidewalk is well connected	3.54 (1.05)
	Pedestrian obstacles	Difficulties due to pedestrian obstacles (standing boards, street vendors)	2.52 (1.03)
Safety	Pedestrian segregation	Most streets segregate pedestrians	3.69 (1.06)
	Crosswalk and traffic lights	Crosswalk and traffic lights are appropriately located	3.81 (0.87)
	Traffic volume	Traffic is heavy on most streets	3.86 (0.91)
	Security facilities	Security facilities (CCTV, street lights, etc.) are sufficiently installed	3.64 (0.92)
Comfort	Green spaces	Streets have sufficient green spaces	3.57 (1.07)
	Natural scenery	Superb natural scenery is available when walking	2.96 (1.22)
	Street cleanness	Little trash on the street and clean	3.16 (1.04)
	Odor and smoke	Difficulty walking due to odors and smokes	2.59 (1.02)
	Noise level	Inconvenience caused by noise from a construction site, vehicle, and residential area	2.85 (1.10)
*Control Variables*			
Individual characteristics			**Frequency (%)**
	Gender	Binary: 0 = male 1 = female	139 (37.5) 232 (62.5)
			**Mean (SD)**
	Age	Continuous: age	34.80 (14.46)
	Weekly minutes of walking	Continuous: minutes	161.62 (115.74)

Note: ^1^ The data is from the survey, measured on a 5-point Likert scale (e.g., 1 = strongly disagree to 5 = strongly agree).

**Table 3:** Built environmental correlates with perceived neighborhood walkability: multiple regression results.

Variables	Model 1				Model 2			
	Unstandardized Coefficients		Standardized Coefficients	t	Unstandardized Coefficients		Standardized Coefficients	t
	B	SE	Beta		B	SE	Beta	
Constant	76.304 ***	11.470	-	6.652	55.625 ***	11.377	-	4.889
Access to amenities used in the Walk Score								
Grocery	2.706	2.434	0.063	1.112	0.632	2.102	0.015	0.301
Restaurants	−5.628	4.125	−0.083	−1.364	−4.375	3.514	−0.065	−1.245
Shopping	2.385	2.635	0.052	0.905	0.705	2.238	0.015	0.315
Coffee shops	−0.733	4.332	−0.011	−0.169	−1.104	3.660	−0.017	−0.302
Banks	3.953	4.036	0.062	0.979	8.223 *	3.526	0.128	2.408
Parks	−0.455	4.755	−0.005	−0.096	−1.966	4.042	−0.023	−0.486
Schools	3.755 *	4.898	0.111	1.992	4.952	4.184	0.056	1.184
Books	−2.599	4.548	−0.035	−0.571	−2.146	3.859	−0.029	−0.556
Entertainment	−0.603	3.168	−0.011	−0.190	−4.550	2.696	−0.082	−1.687
Perception of the built environment								
Hills and stairs					−2.403 **	0.786	−0.149	−3.056
Multiple alternative routes					5.212 ***	0.868	0.289	6.005
Pedestrian segregation					2.248 **	0.849	0.136	2.649
Traffic volume					−0.346	0.880	−0.018	−0.393
Green spaces					2.934 **	0.869	0.179	3.375
Odor and smoke					−1.287	0.822	−0.075	−1.566
Individual characteristics								
Gender (reference: male)					1.399	1.620	0.039	0.863
Age					−0.087	0.055	−0.072	−1.574
Weekly minutes of walking					0.008	0.007	0.054	1.204
R-Squared	0.027				0.335			
Adj-R-Squared	0.003				0.301			
N	371				371			

* *p* < 0.05, ** *p* < 0.01, *** *p* < 0.001.

## Data Availability

The data presented in this study are available on request from the corresponding author.
